# From sequence to function: a new workflow for nitrilase identification

**DOI:** 10.1007/s00253-020-10544-9

**Published:** 2020-04-14

**Authors:** Richard Egelkamp, Ines Friedrich, Robert Hertel, Rolf Daniel

**Affiliations:** Genomic and Applied Microbiology & Göttingen Genomics Laboratory, Institute of Microbiology and Genetics, Georg-August-University of Göttingen, Grisebachstraße 8, 37077 Göttingen, Germany

**Keywords:** Arylacetonitrilase, Metagenome, Nitrilase, Nitrilase assay, Phenylacetonitrile

## Abstract

**Abstract:**

Nitrilases are industrially important biocatalysts due to their ability to degrade nitriles to carboxylic acids and ammonia. In this study, a workflow for simple and fast recovery of nitrilase candidates from metagenomes is presented. For identification of active enzymes, a NADH-coupled high-throughput assay was established. Purification of enzymes could be omitted as the assay is based on crude extract containing the expressed putative nitrilases. In addition, long incubation times were avoided by combining nitrile and NADH conversion in a single reaction. This allowed the direct measurement of nitrile degradation and provided not only insights into substrate spectrum and specificity but also in degradation efficiency. The novel assay was used for investigation of candidate nitrilase-encoding genes. Seventy putative nitrilase-encoding gene and the corresponding deduced protein sequences identified during sequence-based screens of metagenomes derived from nitrile-treated microbial communities were analyzed. Subsequently, the assay was applied to 13 selected candidate genes and proteins. Six of the generated corresponding *Escherichia coli* clones produced nitrilases that showed activity and one unusual nitrilase was purified and analyzed. The activity of the novel arylacetonitrilase Nit09 exhibited a broad pH range and a high long-term stability. The enzyme showed high activity for arylacetonitriles with a *K*_M_ of 1.29 mM and a *V*_max_ of 13.85 U/mg protein for phenylacetonitrile. In conclusion, we provided a setup for simple and rapid analysis of putative nitrilase-encoding genes from sequence to function. The suitability was demonstrated by identification, isolation, and characterization of the arylacetonitrilase.

**Key points:**

• *A simple and fast high-throughput nitrilase screening was developed.*

• *A set of putative nitrilases was successfully screened with the assay.*

• *A novel arylacetonitrilase was identified, purified, and characterized in detail.*

**Electronic supplementary material:**

The online version of this article (10.1007/s00253-020-10544-9) contains supplementary material, which is available to authorized users.

## Introduction

Nitriles are organic compounds that harbor −C≡N as functional group. Many of these compounds are toxic. They are widespread in nature and present in plants as cyanoglycosides (Conn 1979), cyanohydrins in fungi and arthropods, different antibiotics in bacteria, or ricinine and phenylacetonitrile in plants (Jallageas et al. 1980).

For the enzymatic degradation of nitriles, two pathways are known (Supplementary Fig. S1). The first one is the direct conversion of nitriles to corresponding carboxylic acids and ammonia via nitrilases (EC 3.5.5.-). The second one involves the degradation of nitriles to corresponding amides via nitrile hydratases (NHases) (EC 4.2.1.84) and the subsequent hydrolysis of amides to carboxylic acids and ammonia using amidases (EC 3.5.1.4). Respective enzymes are present in bacteria (Egelkamp et al. 2017), filamentous fungi (Martínková et al. 2009), yeasts (Rustler and Stolz 2007), and plants (Piotrowski 2008). The enzymes are used in industry for the production of bulk chemicals such as nicotinic acid or glycolic acid (Shaw et al. 2003; Panova et al. 2007). Novel nitrile-degrading enzymes, especially those with reduced substrate and/or product inhibition under production conditions, are of industrial relevance (Bui et al. 1984; Vaughan et al. 1989; Almatawah and Cowan 1999; Zhang et al. 2011a).

Many nitrile-degrading enzymes were found via (meta)genome mining techniques. A functional screening approach was applied to identify nitrilase-encoding genes from plasmid libraries containing metagenomic DNA from different sources (Robertson et al. 2004; Bayer et al. 2011; Soares Bragança et al. 2017). In addition, in silico screening of (meta)genomic sequence data was used to identify putative nitrilases and nitrile hydratases in publicly available databases (Vergne-Vaxelaire et al. 2013). Although sequence-based approaches lead very quickly to many new gene candidates for the targeted enzyme type, they remain as predictions until functional verification. To overcome this limitation and take advantage of the constantly growing metagenomic sequence pool, procedures are needed for efficient verification of nitrilase activity and identification of potential substrates of these enzymes.

The focus of this study was to establish a workflow that enables the fast functional verification of sequence-based, screening-derived putative nitrilases and their substrates. Six metagenomes, which were obtained during a study targeting the impact of different nitriles on microbial communities (Egelkamp et al. 2019), were chosen as starting material. A procedure was established to reduce the 70 putative nitrilase-encoding genes that had been inferred from sequence-driven mining of the metagenomes to candidates with verified enzyme activity. Serving as proof of concept, one enzyme candidate was heterologously expressed and purified. Subsequently, the recovered enzyme and its activity were characterized, including the determination of optimal pH, temperature range, stability, substrate specificity, and enzyme kinetics. In addition, influences of divalent ions and other substances on enzyme activity were determined.

## Material and methods

### Origin of nitrilases

Putative nitrilase-encoding genes were identified in, and amplified from, metagenomic DNA that originated from compost-derived enrichment cultures containing either phenylacetonitrile, succinonitrile, acetonitrile, crotononitrile, 4-hydroxybenzonitrile, or cyclohexanecarbonitrile (Egelkamp et al. 2019). The compost sample was collected in the Experimental Botanical Garden of Georg-August-Universität Göttingen (Germany; 51° 33′ 22.6″ N, 9° 57′ 16.2″ E). GenBank accession numbers of the annotated corresponding 6 metagenomes are as follows: phenylacetonitrile, RCUE00000000; succinonitrile, RCUP00000000; acetonitrile, RCUQ00000000, crotononitrile, RCUN00000000; 4-hydroxybenzonitrile, RCUF00000000, and cyclohexanecarbonitrile, RCUO00000000.

An already characterized aliphatic nitrilase from *Rhodococcus rhodochrous* K22 was used as positive control for establishing the high-throughput nitrilase assay (Kobayashi et al. 1990; Kobayashi et al. 1992b). For this purpose, the corresponding nitrilase gene was codon-optimized for *Escherichia coli* K12 derivates using the web suite JCat (http://www.jcat.de) (Grote et al. 2005) and synthesized by Integrated DNA Technologies (Leuven, Belgium).

### Bioinformatic analysis of described and putative nitrilases

Nitrilase sequence reference data were obtained from the SWISS-Prot database (Bairoch and Apweiler 2000; date of search: October 14, 2018). The data were further processed by removing misannotated enzymes, mere subunits, or nitrile hydratases designated as nitrilases. Clustering of data was done with CD-HIT (Huang et al. 2010). A fasta file containing the SWISS-Prot nitrilases and metagenome-deduced nitrilases was used as input and a sequence identity cutoff of 40% was set for clustering (Supplementary Data File S1).

### Growth medium

Lysogeny broth (LB) (10 g tryptone, 10 g NaCl, and 5 g yeast extract per liter) was used for growth of microorganisms. For solid media, 15 g agar per liter was added.

### Amplification of metagenome-encoded putative nitrilases

PCR reaction mixture (total volume 50 μL) contained 10 μL 5-fold Phusion HF buffer, 200 μM of each dNTP, 0.2 μM of each primer (Supplementary Table S1), 3% DMSO, 50 ng metagenomic DNA as template and 1 U of Phusion polymerase (Thermo Fisher Scientific, Waltham, MA, USA). Initial denaturation was performed at 98 °C for 5 min, followed by 30 cycles of denaturation at 98 °C for 30 s, annealing (temperature based on primer melting temperature) for 30 s and elongation at 72 °C for 45 s per kbp. The final elongation was for 5 min at 72 °C.

### Plasmids, strains, and transformation

Amplified putative nitrilase-encoding genes (Supplementary Data File S2) were cloned into the pBAD18 vector system (Guzman et al. 1995). Chemically competent *E. coli* TOP10 cells were transformed according to the protocol of the manufacturer (Thermo Fisher Scientific, Waltham, MA, USA). Subsequently, cells were plated on 100 μg/mL ampicillin-containing LB plates and incubated overnight at 37 °C. The fidelity of the constructs was checked by Sanger sequencing (Microsynth Seqlab, Göttingen, Germany).

### Heterologous expression of putative nitrilases and purification of His_6_-tagged proteins

*E. coli* TOP10 strains containing the pBAD18-based recombinant plasmids were grown in LB medium at 180 rpm (Innova 44 shaker, New Brunswick Scientific, Nürtingen, Germany) and 37 °C overnight. The preculture was used to inoculate 10 mL LB medium with an OD_600_ of 0.1. Subsequently, the culture was incubated for 1.5 h at 37 °C and 180 rpm (Innova 44 shaker) to a final OD_600_ of 0.6–0.8. For induction of heterologous gene expression, 1.5% l-(+)-arabinose was added, followed by 6 h of incubation.

Cells containing the produced (His_6_-tagged) nitrilase were washed twice with 1× LEW buffer of the Protino Ni-TED kit (Macherey-Nagel, Düren, Germany) and resuspended in 1.5 mL of the same buffer containing 40 μg/mL DNase I and 0.1% (w/v) lysozyme. The cells were then disrupted by at least three passages through a French press at 1.38 × 10^8^ Pa (Thermo Fisher Scientific). The extract was cleared by centrifugation at 6000×*g* and 4 °C for 20 min. The recovered supernatant was loaded onto Protino Ni-TED columns according to the protocol of the manufacturer (Macherey-Nagel). The purified enzyme was further analyzed by 12% sodium dodecyl sulfate-polyacrylamide gel electrophoresis (SDS-PAGE) (Laemmli 1970). Protein concentration was determined using the Bradford method (Bradford 1976) with bovine serum albumin as standard.

### Substrates for nitrilase screenings

Stock solutions of nitriles (final concentration of 2.7 M) were generated by solving the nitriles in *N*,*N*-dimethylformamide (DMF) (Merck KGaA, Darmstadt, Germany). These stock solutions were sterile-filtered and stored at 4 °C. The following nitriles were used: phenylacetonitrile, acetonitrile (both TCI Deutschland GmbH, Eschborn, Germany), succinonitrile, crotonitrile, 4-hydroxybenzonitrile, acetone cyanohydrin, cyclohexanecarbonitrile, fumaronitrile, and 2-phenylpropionitrile (all Sigma-Aldrich Chemie GmbH, Munich, Germany). In addition, mandelonitrile, 2-phenylbutyronitrile, benzonitrile, 3-indoleacetonitrile, 2-thiopheneacetonitrile, 3-thiopheneacetonitrile, (2-chlorophenyl)acetonitrile, (3-chlorophenyl)acetonitrile, (4-chlorophenyl)acetonitrile, cinnamonitrile, 1,4-phenylenediacetonitrile, 2-naphtylacetonitrile (all Sigma-Aldrich), and 3-phenylpropiontrile (Alfa Aesar, Haverhill, MA, USA) were used for further characterization of the purified arylacetonitrilase.

### High-throughput nitrilase activity assay

Degradation of nitriles was measured by monitoring the release of ammonia in a coupled enzymatic reaction (Reisinger et al. 2006). The ammonia reacts with NADH and α-ketoglutarate in the presence of a glutamate dehydrogenase from bovine liver type II (GDH; Sigma-Aldrich) to glutamate. The reduced cofactor is monitored by its absorbance change at 340 nm. For screening of putative nitrilases, the high-throughput assay of Vergne-Vaxelaire (Vergne-Vaxelaire et al. 2013) was adjusted to allow real-time measurements without time-consuming incubation steps. The assay was performed in a flat-based 96-well plate. The reaction mixture (final volume 250 μL) contained 0.5 mM NADH, 1 mM α-ketoglutarate, 37 μL of a 2.7-M nitrile stock solution (solved in DMF), 20 μg crude extract (added at the end), 1 U/mL GDH, and 50 mM Tris-HCl (pH 8.0) buffer. Crude extract containing the pBAD18 cloning vector without insert was used as negative control. The reaction mixture was incubated at 37 °C in a Synergy 2 microplate reader (BioTek Instruments GmbH, Bad Friedrichshall, Germany). The absorbance was constantly measured for 1.5 h at 340 nm. For calculation of enzyme activity, the values of the pBAD18 crude extract (negative control) were subtracted from that of the crude extract containing putative nitrilases. The activity of the nitrilase from *R. rhodochrous* K22 (positive control) with different substrates was described previously (Kobayashi et al. 1990; Kobayashi et al. 1992b). During the first 15 min of measurement using the control enzyme, the degradation of succinonitrile, fumaronitrile, and crotononitrile was observed and the absorbance decrease was at least 0.75. Thus, only reactions completed during the first 15 min of measurement and showing at least an absorbance decrease of 0.75 were considered as nitrilase activity and selected for the subsequent analyses.

### Construction of a phylogenetic tree

For calculation of a phylogenetic nitrilase tree, MEGA X version 10.1.7 (Kumar et al. 2018) was used. Fifteen characterized nitrilases and 3 amidases were recovered from the NCBI database and used as references. Arylacetonitrilases were derived from *Alcaligenes faecalis* JM3 (D13419; Kobayashi et al. 1993), *Pseudomonas putida* MTCC 5110 (EF467660; Banerjee et al. 2009), and *Pseudomonas fluorescens* EBC191 (AY885240; Kiziak et al. 2005). For aliphatic nitrilases, the enzymes from *Rhodococcus rhodochrous* K22 (D12583; Kobayashi et al. 1992b), *Comamonas testosteroni* (L32589; Lévy-Schil et al. 1995), and *Synechocystis* sp. PCC6803 (BA000022; Heinemann et al. 2003) were used as references. Sequences for aromatic nitrilases were obtained from *Rhodococcus rhodochrous* J1 (D11425; Kobayashi et al. 1992a), *R. rhodochrous* NCIMB 11216 (AX235749; Ress-Löschke et al. 2001), and *Aeribacillus pallidus* RAPc8 (DQ826045; Williamson et al. 2010). References for cyanide dihydratases were taken from *Pseudomonas stutzeri* AK61 (KM459551; Crum et al. 2015), *Bacillus pumilus* C1 (AF492815; Jandhyala et al. 2003), and *B. pumilus* 8A3 (AF492814; Jandhyala et al. 2003). β-Cyano-l-alanine nitrilases originated from *Pseudomonas protegens* Pf-5 (CP000076; Paulsen et al. 2005; Howden et al. 2009), *P. fluorescens* Pf0-1 (CP000094; Howden et al. 2009; Silby et al. 2009), and *Pseudomonas pseudoalcaligenes* CECT 5344 (HG916826; Wibberg et al. 2014; Acera et al. 2017). In addition, amidases from *R. rhodochrous* M8 (AY971668; Riabchenko et al. 2006), *P. aeruginosa* PAO1 (AE004091; Ambler et al. 1987), and *P. fluorescens* SBW25 (AM181176; Howden et al. 2009; Silby et al. 2009) were included. The reference sequences and 6 putative nitrilases identified in this study were aligned with MUSCLE (Edgar 2004), leading to 1401 positions in the final dataset. Subsequently, evolutionary analysis was performed using the Maximum Likelihood method with the General Time Reversible model (Nei and Kumar 2000) and a “very weak” branch swap filter. Evolutionary rate differences among sites were modeled with a discrete Gamma distribution (5 categories; +G, parameter = 2.0817). The tree with the highest log likelihood (− 20,729.17) is shown. Test of phylogeny was performed by calculation of 1000 bootstrap replicates, and nodes with values below 50% were condensed for the final image.

### Nitrilase characterization

The activity of a novel arylacetonitrilase (Nit09) was characterized by using a colorimetric assay for detection of ammonia (Fawcett and Scott 1960). As the reagents are temperature-sensitive, all experiments were performed in an air-conditioned laboratory at 23 °C. Nitrilase reaction was performed in a 500-μL mixture containing 25 μL nitrile (100 mM solved in DMF), 1.25 μg purified His_6_-tagged nitrilase, and 0.1 M citrate-phosphate buffer (pH 6.0) for 2 min at the temperature optimum of 50 °C. Then, 111 μL of this reaction was transferred into 222 μL sodium phenoxide (0.266 M phenol and 4 M NaOH), followed by 333 μL of 0.01% sodium nitroprusside and 333 μL of 0.02 N hypochlorite. Ammonia reacts with sodium phenoxide and hypochlorite to produce indophenol blue. Sodium nitroprusside improves the intensity, reproducibility, and stability of the blue color (Kaplan et al. 1965). The reaction mixture was incubated for 15 min at 27 °C in darkness. Subsequently, absorption was measured at 630 nm. To determine the substrate spectrum of Nit09, 20 mM of the tested nitrile was used and the reaction mixture was incubated for 15 min.

### Nitrilase inhibitors and enzyme stability

Effects of different potential inhibitors (EDTA, DTT, H_2_O_2_, HgCl_2_, AgNO_3_, CaCl_2_, MnSO_4_, MgSO_4_, FeSO_4_, CuSO_4_, CoSO_4_, ZnCl_2_, NaN_3_, and SDS) and solvents (methanol, ethanol, glycerol, isopropanol, DMSO, acetone, chloroform, and toluene) on Nit09 activity were tested. Incubation was carried out at 50 °C for 2 min in the standard reaction mixture containing a putative inhibitor at 1 mM or 5% (v/v) and 20% (v/v) of tested organic solvent. For stability analysis, the His_6_-tagged purified Nit09 was stored in Protino® Ni-TED elution buffer (50 mM NaH_2_PO_4_, 300 mM NaCl, 250 mM imidazole, pH 8.0) containing 1 mM NaN_3_ at 4 °C in the dark for 91 days. Enzymatic activity was determined under standard reaction conditions.

### Determination of enzymatic constants

The steady-state kinetics for Nit09 activity was measured in the presence of GDH to detect the concomitant oxidation of NADH at 340 nm and 37 °C using a UV/Vis spectrophotometer (Cary 100 UV-Vis, Varian Medical Systems, Darmstadt, Germany). The reaction mixture (final volume 1 mL) contained 0.5 mM NADH, 1 mM α-ketoglutarate, 0.1 mM to 100 mM of phenylacetonitrile (final volume 37 μL), 5 μg purified nitrilase Nit09, 1 U/mL GDH from bovine liver type II (Sigma-Aldrich), and 0.1 M HEPES buffer (pH 8.0). Negative controls were prepared without nitrilase. To determine the turnover number and the maximum number of chemical conversions of phenylacetonitrile molecules per second that a single catalytic site will execute for a given enzyme concentration, the slope (∆A_340_) had to be first determined. This was done by the software of Cary WinUV version 3.0 (Varian Medical Systems). With the slope, the *k*_cat_ values were determined and plotted against phenylacetonitrile concentrations. Further, the constants *k*_cat_ and *K*_M_ were analyzed using the Michaelis-Menten equation of the R package “drc” (Ritz et al. 2015).

## Results

### Optimization of the NADH-coupled nitrilase assay

**Fig. 1:**
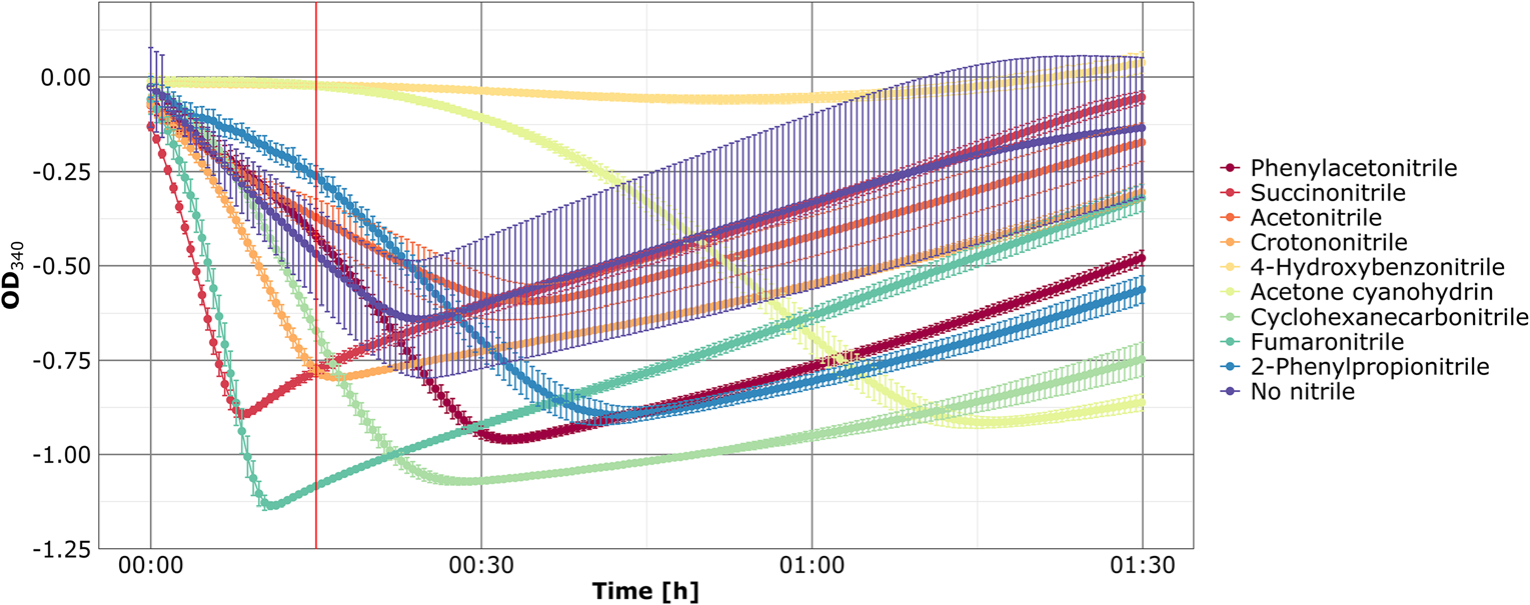
Nitrile degradation by the *R. rhodochrous* K22 nitrilase. The assay was performed in triplicate at 37 °C in a 96-well microtiter plate. NADH conversion was monitored at 340 nm. Fumaronitrile, succinonitrile, and crotononitrile were degraded after 15 min, thereby defining the threshold (red line) for the required experimental time

Most current high-throughput nitrilase assays have drawbacks such as low sensitivity (Yazbeck et al. 2006), the requirement of specific hardware like fluorometers (Banerjee et al. 2003), or suitability only for specific nitriles (Zhu et al. 2007b). A promising approach is a NADH-coupled assay in which ammonia released by the nitrilase reaction is consumed by a glutamate dehydrogenase (GDH) in the course of the conversion of NADH to NAD. The conversion of this cofactor is monitored at 340 nm and reveals activity of the tested (putative) nitrilases (Vergne-Vaxelaire et al. 2013). However, it requires extended incubation times for nitrilase reaction and provides only qualitative information and no insights into enzymatic efficiency. We modified the assay to allow fast and simple screenings of nitrilase activity with different substrates without additional incubation steps in a single reaction mixture. Therefore, concentrations of all test ingredients were optimized to facilitate continuous measurement of NADH conversion in a 96-well microtiter plate providing first insights into the efficiency of nitrile degradation. In comparison to the method of Vergne-Vaxelaire (Vergne-Vaxelaire et al. 2013), the amount of NADH was increased to 0.5 mM for a larger measurement range and the concentration of α-ketoglutarate was reduced to prevent inhibition of the GDH reaction. These modifications were tested using crude extract containing an already characterized nitrilase from *Rhodococcus rhodochrous* K22 (Kobayashi et al. 1990; Kobayashi et al. 1992b) as proof of principle (Fig. 1).

To minimize the identification of false positives or enzymes with low activity, two thresholds were defined. During the first 15 min of measurement, total conversion of succinonitrile, fumaronitrile, and crotononitrile by the K22 control nitrilase was recorded and the absorbance decrease at 340 nm was at least 0.75. As K22 nitrilase activity for these substrates was described previously (Kobayashi et al. 1990; Kobayashi et al. 1992b), only reactions active in the first 15 min and with an absorbance decrease of at least 0.75 were considered as positive for substrate conversion and nitrilase activity during subsequent experiments.

### Screening of putative metagenome–derived nitrilase-encoding genes and selection of candidates for activity analysis of the corresponding enzymes

Six metagenomes of microbial communities individually obtained from phenylacetonitrile-, succinonitrile-, acetonitrile-, crotononitrile-, 4-hydroxybenzonitrile-, or cyclohexanecarbonitrile-containing enrichment cultures (Egelkamp et al. 2019) served as sources for putative nitrilases. In total, 70 annotated putative nitrilase-encoding genes were identified in these metagenomes (Supplementary Data File S2). In a first step, the sequences of the identified genes and the corresponding deduced proteins were analyzed (Supplementary Data File S2). Initially, to relate the deduced protein sequences with already characterized enzymes, all deduced protein sequences of the metagenome-derived nitrilase candidates were compared with the amino acid sequences of 46 characterized reference nitrilases obtained from SWISS-Prot. This analysis resulted in 13 nitrilase protein clusters (Supplementary Data File S3) and reduced our data set to 60 unique candidates. The first three clusters contained characterized nitrilases obtained from SWISS-Prot as well as our putative metagenome-derived nitrilases with molecular masses ranging from 5.96 to 38.68 kDa (cluster 1), 12.7 to 40.2 kDa (cluster 2), and 10.41 to 38.62 kDa (cluster 3). Clusters 4, 6, 10, 11, 12, and 13 consisted solely of characterized SWISS-Prot-derived nitrilases, whereas the remaining four clusters (5, 7, 8 and 9) included only our putative metagenome-derived enzyme sequences. As the smallest characterized prokaryotic nitrilase originates from *Pyrococcus abyssi* GE5 and consists of 262 amino acids (Mueller et al. 2006), all putative nitrilases smaller than this were checked for the presence of ribosomal binding sites (RBS) in the corresponding metagenomic DNA region to exclude artificial peptides caused by incomplete metagenomic assembly or misassembly. Finally, 37 candidates were selected for cloning and overexpression in *E. coli*.

After amplification of the respective genes from metagenomic DNA and sequence verification, frameshift mutations were observed in four cases, leading to their exclusion. In 31 cases, variations resembling point mutations of the metagenome-derived gene sequences were recorded. Probably, the sequencing depth of the metagenomes was not sufficient to distinguish between very similar sequences, leading to the amplification of previously unidentified gene variants. All of these variations were included in subsequent analyses. In total, 26 putative nitrilase-encoding genes were successfully subcloned (Supplementary Data File S2). Analysis of heterologous protein formation revealed production of 13 putative nitrilases by the *E. coli* host. Although the remaining 13 putative nitrilases could be successfully overexpressed, the corresponding enzymes were not present in the cleared cell-free crude extract. These enzymes were detected in the pellet that remains after cell disruption and clearing the crude extract by centrifugation. These enzymes were not considered for subsequent analysis, as we assumed that these proteins were part of inclusion bodies.

**Fig. 2:**
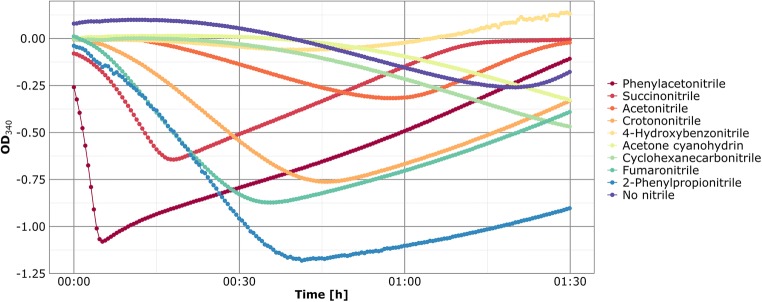
Substrate specificity of Nit09. The assay was performed at 37 °C in a 96-well plate. NADH conversion was monitored at 340 nm. Degradation of phenylacetonitrile was followed until the previously determined 15 min threshold (red line)

The previously established assay was used to verify nitrilase activity with the 13 putative nitrilase candidates using nine different nitriles as substrate (Table 1). Six of the putative enzymes showed nitrilase activity with at least one nitrile. Most were active with succinonitrile or fumaronitrile. In addition, one of these (Nit09) could act on the aromatic phenylacetonitrile (Fig. 2).

**Table 1:** Origin and substrate specificity of expressed putative nitrilases

Nitrilase	Metagenomic origin^a^	Size (bp)/mass (kDa)	Closest relative^b^	Substrate specificity^c^	NCBI accession number
Nit09	SUN	1005/36.12	*Variovorax boronicumulans* J1 nitrilase (Q: 100%, E: 0.0, I: 98.1%, A: KY937903)	PAN	MN689843
Nit10	SUN	924/33.00	*Pseudomonas fluorescens* NCIMB 11764 nitrilase (Q: 99%, E: 0.0, I: 87.7%, A: CP010945)	–	MN689844
Nit14	SUN	975/35.13	*Pseudomonas* sp. S34 aliphatic nitrilase (Q: 100%, E: 0.0, I: 96.3%, A: CP019398)	SUN, FUN	MN689845
Nit24	ACN	1068/38.70	*Janthinobacterium* sp. Marseille nitrilase (Q: 99%, E: 0.0, I: 86.9%, A: CP000269)	–	MN689846
Nit28	ACN	1059/38.62	*Pseudomonas* sp. LAB-08 nitrilase (Q: 97%, E: 0.0, I: 88.9%, A: AP017423)	SUN, FUN	MN689847
Nit30	ACN	924/32.93	*Pseudomonas fluorescens* NCIMB 11764 nitrilase (Q: 99%, E: 0.0, I: 87.4%, A: CP010945)	–	MN689848
Nit37	ACN	1017/36.74	*Cupriavidus basilensis* 4G11 nitrilase (Q: 98%, E: 0.0, I: 86.7%, A: CP010537)	SUN, FUN	MN689849
Nit56	CRN	975/35.20	*Pseudomonas* sp. UW4 nitrilase (Q: 100%, E: 0.0, I: 97.4%, A: CP003880)	SUN, FUN	MN689850
Nit57	CRN	975/35.18	*Pseudomonas* sp. UW4 nitrilase (Q: 100%, E: 0.0, I: 98.0%, A: CP003880)	–	MN689851
Nit59	CRN	924/32.84	*Pseudomonas* sp. UW4 nitrilase (Q: 100%, E: 0.0, I: 95.1%, A: CP003880)	–	MN689852
Nit60	CRN	924/32.86	*Pseudomonas* sp. S34 nitrilase (Q: 100%, E: 0.0, I: 98.6%, A: CP019398)	FUN	MN689853
Nit66	CRN	924/32.91	*Pseudomonas* sp. UW4 nitrilase (Q: 100%, E: 0.0, I: 97.4%, A: CP003880)	–	MN689854
Nit78	CCN	924/32.83	*Pseudomonas* sp. S34 nitrilase (Q: 100%, E: 0.0, I: 99.2%, A: CP019398)	–	MN689855

^a^Metagenomic origin of putative nitrilase genes. SUN, succinonitrile treatment; ACN, acetonitrile treatment; CRN, crotononitrile treatment; CCN, cyclohexanecarbonitrile treatment

^b^Gene sequences were used for a blastn search against the NCBI nr database and best hits are shown. Q, query cover; E, e value; I, identity; A, accession

^c^Substrate specificity determined with high-throughput assay. Tested substrates were PAN, phenylacetonitrile; SUN, succinonitrile; ACN, acetonitrile; CRN, crotononitrile; HBN, 4-hydroxybenzonitrile; ACH, acetone cyanohydrin; CCN, cyclohexanecarbonitrile; FUN, fumaronitrile; PPN, 2-phenylpropionitrile; −, no enzymatic activity with tested substrates

**Fig. 3:**
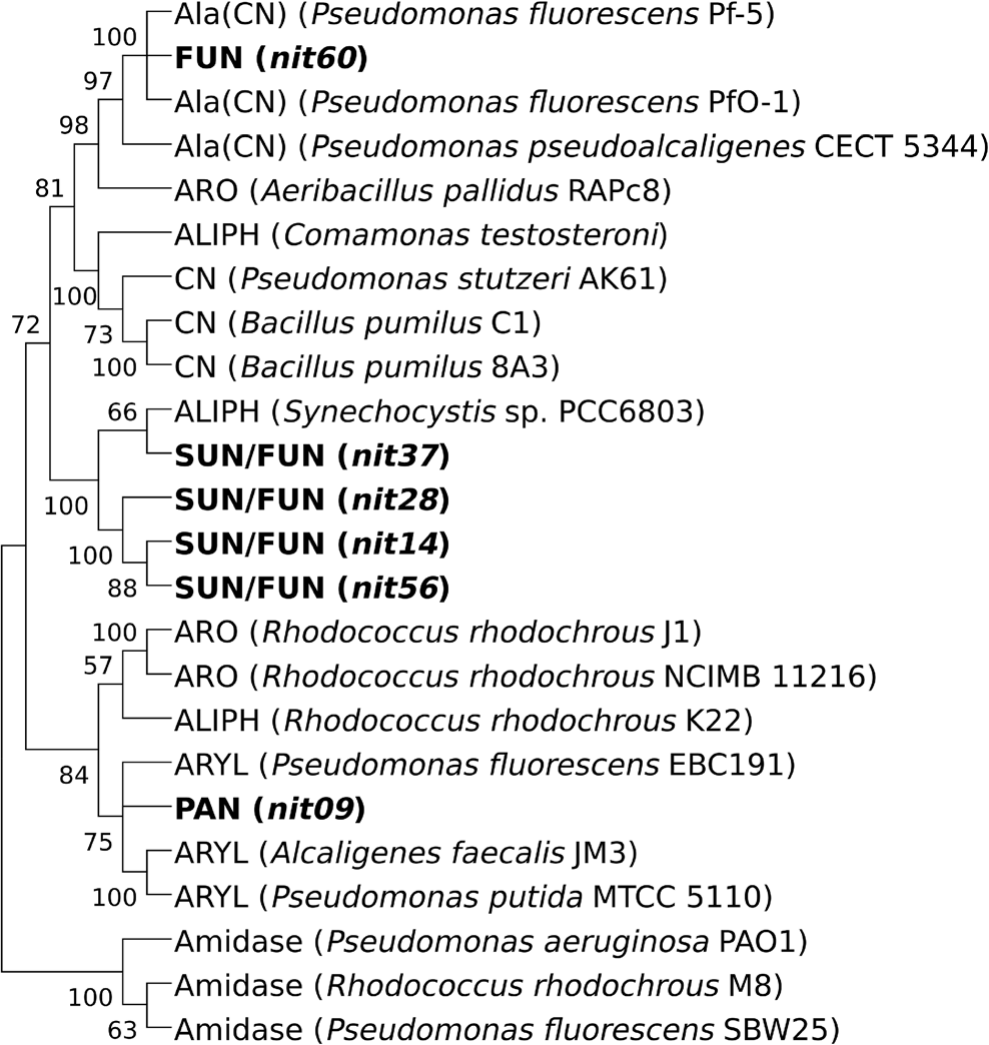
Phylogenetic nitrilase tree. Metagenomic sequences used in this study are in bold letters. PAN phenylacetonitrile, SUN succinonitrile, FUN fumaronitrile, ARYL arylacetonitrilase, ALIPH aliphatic nitrilase, ARO aromatic nitrilase, CN cyanide dihydratase, Ala(CN) β-cyano-l-alanine nitrilase

Gene sequences of the six active enzymes and already characterized reference nitrilases were used to calculate a phylogenetic tree (Fig. 3). The phenylacetonitrile-specific nitrilase (Nit09) clustered with arylacetonitrilases, whereas the four nitrilases active with succinonitrile and fumaronitrile (Nit14, Nit28, Nit37, and Nit56) form a branch with an aliphatic nitrilase from *Synechocystis* sp. PCC6803, and the fumaronitrile-degrading nitrilase (Nit60) most closely resembled with three β-cyano-l-alanine nitrilases.

### Characterization of arylacetonitrilase Nit09

Phenylacetonitrile belongs to the class of arylacetonitriles, a subclass of nitriles degraded by the rare group of arylacetonitrilases (EC 3.5.5.5). We chose the phenylacetonitrile-degrading enzyme candidate Nit09 for detailed characterization and verification of enzymatic activity under defined conditions as it represented probably a novel arylacetonitrilase. This assumption was further supported by the clustering of *nit09* with other arylacetonitrilases in the previously calculated phylogenetic tree (Fig. 3). The His_6_-tagged enzyme was produced and purified by immobilized metal ion affinity chromatography. SDS-PAGE analysis of the enzyme preparation confirmed production of a 36 kDa enzyme, which is in accordance with the Nit09 molecular mass deduced from the gene sequence (data not shown). Phenylacetonitrile was used as substrate to further characterize the purified enzyme, since Nit09 showed the ability to degrade phenylacetonitrile in the high-throughput screening.

**Fig. 4:**
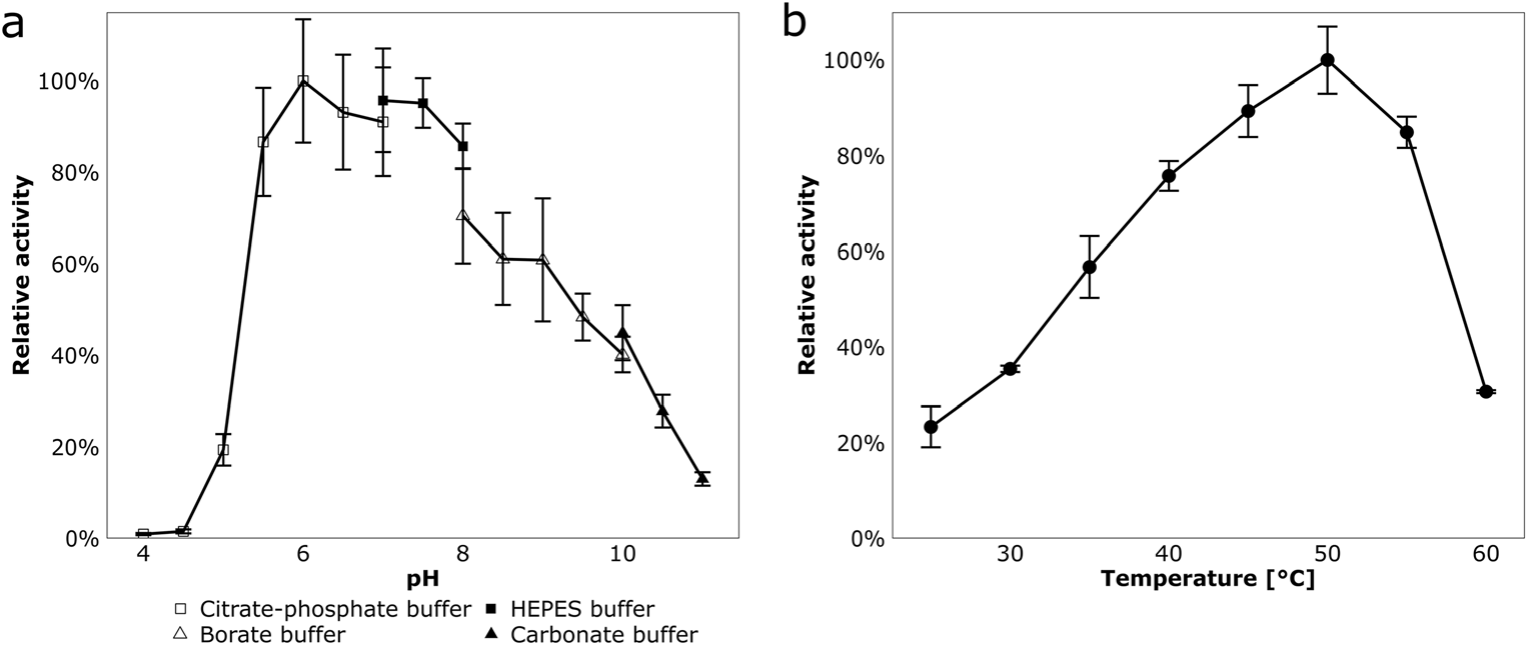
Dependency of Nit09 activity on pH (**a**) and temperature (**b**). **a** Reactions were performed for 2 min at 37 °C in the respective buffers (all 0.1 M final concentration) containing 1.25 μg purified protein and 5 mM phenylacetonitrile. One hundred percent relative activity corresponds to 2.79 U/mg. **b** Reactions were performed for 2 min at various temperatures using citrate-phosphate buffer (pH 6.0), 1.25 μg purified protein, and 5 mM phenylacetonitrile. One hundred percent relative activity corresponds to 5.68 U/mg

**Fig. 5:**
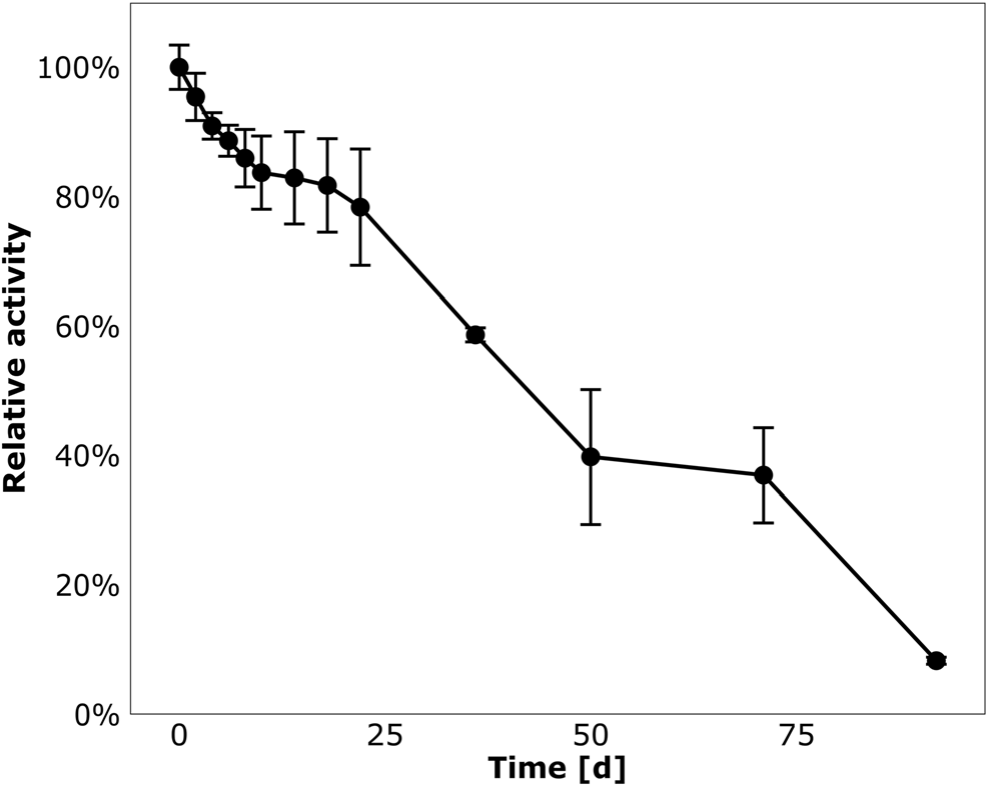
Stability assay with Nit09. Enzyme was stored for 91 days at 4 °C in a buffered system (50 mM NaH_2_PO_4_, 300 mM NaCl, 250 mM imidazole, 1 mM NaN_3_, pH 8.0). Reactions were carried out for 2 min at 50 °C using citrate-phosphate buffer (pH 6.0), 1.25 μg purified protein, and 5 mM phenylacetonitrile. One hundred percent relative activity corresponds to 5.68 U/mg

Enzyme activity was highest at pH 6 with approximately 90% activity being retained between pH 5.5 and 8. At both pH 5 and pH 10.5, the relative activity dropped to 20% (Fig. 4a). Optimum temperature of enzyme activity was at 50 °C, whereas nitrilase activity was reduced to 30% at 60 °C (Fig. 4b). Furthermore, stability of the nitrilase was tested over several weeks under storage conditions at 4 °C. Remaining catalytic activity of > 80% was observed during the first 3 weeks, followed by a slow decline (Fig. 5). After 3 months, 8% of the initial activity remained.

To further examine the substrate spectrum, the Nit09 nitrilase was initially tested with the same substrates used for the high-throughput assay. In this way, the results of the above-described screening assay were verified and phenylacetonitrile was the only one of these substrates converted by Nit09 under the tested conditions (Table 2). To determine activity of the enzyme with structurally related compounds, 13 aromatic nitriles were additionally tested. With four of them (2-phenylbutyronitrile, benzonitrile, 2-naphtylacetonitrile, and cinnamonitrile), nitrilase activity was not detected. With 2-chlorophenylacetonitrile, 3-chlorophenylacetonitrile, and 4-chlorophenylacetonitrile as substrates, only low relative activities (5 to 12%) were recorded compared to phenylacetonitrile. Reduced relative activity was also detected for 3-phenylpropionitrile (16%), 1,4-phenylenediacetonitrile (19%,) mandelonitrile (22%), and 3-indoleacetonitrile (34%). Strikingly, significantly higher relative activity was detected for 3-thiopheneacetonitrile (188%) and 2-thiopheneacetonitril (385%).

**Table 2:** Substrate specificity of Nit09. Reactions were run for 15 min at 50 °C using 0.1 M citrate-phosphate buffer (pH 6.0), 1.25 μg purified protein, and 20 mM nitrile

Substrate	Relative activity (%)^a^
Phenylacetonitrile	100.00 ± 1.19
Succinonitrile	0.17 ± 0.03
Acetonitrile	0.00 ± 0.01
Crotononitrile	0.08 ± 0.01
4-Hydroxybenzonitrile	0.00 ± 0.00
Acetone cyanohydrin	0.13 ± 0.02
Cyclohexanecarbonitrile	0.09 ± 0.01
Fumaronitrile	0.45 ± 0.04
2-Phenylpropionitrile	0.52 ± 0.05
Mandelonitrile	22.01 ± 0.61
2-Phenylbutyronitrile	0.07 ± 0.01
3-Phenylpropionitrile	15.81 ± 0.80
Benzonitrile	0.48 ± 0.32
2-Naphthylacetonitrile	0.11 ± 0.00
1,4-Phenylenediacetonitrile	18.82 ± 0.62
2-Chlorophenylacetonitrile	11.69 ± 0.80
3-Chlorophenylacetonitrile	5.34 ± 0.06
4-Chlorophenylacetonitrile	4.95 ± 0.17
Cinnamonitrile	0.12 ± 0.01
3-Indoleacetonitrile	34.38 ± 0.78
2-Thiopheneacetonitrile	385.46 ± 0.78
3-Thiopheneacetonitrile	187.53 ± 5.75

^a^Activity with phenylacetonitrile as substrate (5.19 U/mg) was set as 100% relative activity

To assess the effect of potential inhibitors and solvents on enzyme activity, the activity of the purified enzyme was tested in the presence of different chemicals. For detection of thiol residues essential for enzyme activity, the thiol-binding reagents HgCl_2_ and AgNO_3_ were applied to the reaction mixture. The recorded decline of enzyme activity to 1.6 and 2.0%, respectively, indicated that the functionality of Nit09 depends on these residues (Table 3). As the metal-chelating agent EDTA had no significant effect on enzymatic activity, dependence on divalent metal cofactors is not indicated. This was further supported by the lack of any effect when divalent ions such as Mg^2+^, Fe^2+^, and Mn^2+^ were added to the reaction mixtures. Only addition of Zn^2+^ resulted in a slight relative activity increase to 114%.

**Table 3:** Effects of various compounds on the activity of Nit09. Reactions were run for 2 min at 50 °C using 0.1 M citrate-phosphate buffer (pH 6.0), 1.25 μg purified protein, 5 mM phenylacetonitrile, and 1 mM putative inhibitor

Compound	Relative activity (%)^a^
Without additives	100.00 ± 0.64
EDTA	104.45 ± 1.62
DTT	76.21 ± 3.29
H_2_O_2_	24.56 ± 2.35
HgCl_2_	1.57 ± 0.36
AgNO_3_	1.96 ± 0.18
CaCl_2_	106.29 ± 2.71
MnSO_4_	98.01 ± 3.80
MgSO_4_	101.87 ± 5.92
FeSO_4_	98.25 ± 5.65
CuSO_4_	86.98 ± 3.82
CoSO_4_	81.01 ± 2.60
ZnCl_2_	114.00 ± 2.04
Sodium azide	98.89 ± 2.13
SDS	12.02 ± 2.77

^a^Activity with phenylacetonitrile as substrate without additional additives (5.68 U/mg) was set as 100% relative activity

Strong inhibitory effects were encountered in the presence of DMSO, acetone, and chloroform even at 5% concentration (Table 4). At this concentration, the nitrilase was less sensitive to methanol, ethanol, isopropanol, and glycerol treatment. At 20% concentration, only glycerol did not abolish enzymatic activity.

**Table 4:** Effect of different organic solvents on the activity of Nit09. Reactions were run for 2 min at 50 °C using 0.1 M citrate-phosphate buffer (pH 6.0), 1.25 μg purified protein, and 5 mM phenylacetonitrile

Solvent	Relative activity with 5% v/v organic solvent (%)^a^	Relative activity of enzyme with 20% v/v organic solvent (%)^a^
Without solvent	100.00 ± 7.49	100.00 ± 7.49
Methanol	88.01 ± 6.18	9.26 ± 1.97
Ethanol	85.22 ± 7.12	4.82 ± 0.41
Glycerol	81.11 ± 8.68	76.65 ± 9.06
Isopropanol	77.55 ± 9.43	3.63 ± 0.91
DMSO	4.80 ± 1.06	2.32 ± 0.72
Acetone	28.96 ± 4.79	0.14 ± 0.07
Chloroform	24.81 ± 3.44	3.70 ± 1.38
Toluene	8.12 ± 2.13	2.44 ± 1.60

^a^Activity with phenylacetonitrile as substrate without additional additives (5.68 U/mg) was set as 100% relative activity

**Fig. 6:**
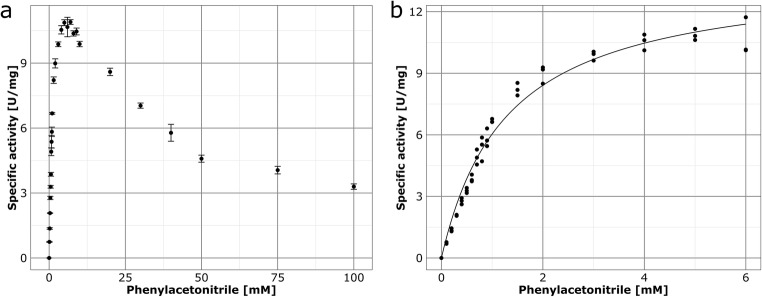
Influence of substrate concentrations on the reaction rate of Nit09. Reactions were performed with phenylacetonitrile as substrate under standard conditions at 37 °C. **a** Steady-state measurement until 100 mM substrate concentration. **b** Steady-state measurement until saturation point at 6 mM substrate concentration

Finally, kinetic constants of Nit09 were determined. The reaction rate increased with phenylacetonitrile concentration until a saturation was reached at 6 mM. Between 10 and 50 mM, a rapid decrease of activity was recorded, followed by a slow decline up to 100 mM (Fig. 6a), indicating inhibition by the substrate. Based on these data, kinetic constants were calculated with a non-linear model due to superior precision compared to linear methods like Lineweaver-Burk plots (Cho and Lim 2018). Phenylacetonitrile was converted at 37 °C with a *K*_M_ of 1.29 mM and *V*_max_ of 13.85 U/mg, reaching the saturation point at 6 mM (Fig. 6b).

## Discussion

### From putative nitrilase sequences to nitrilase function

In this study, a workflow and a fast and simple assay for screening of functional nitrilases was established. In comparison to the previously described method (Vergne-Vaxelaire et al. 2013), it reduces the total screening time from approx. 5 h to approximately 45 min by combining nitrilase reaction and subsequent ammonia conversion in a single reaction. The use of crude extract is less time-consuming compared to methods demanding purified enzymes. Furthermore, the assay system allows continuous measurement of nitrile degradation and provides insights into the efficiency and substrate spectra of the tested enzymes. However, its precision is limited by the GDH as only nitrilase reactions slower than that of GDH can be monitored in detail. In addition, other enzymes in the crude extract might cause a NAD release or regeneration of NAD to NADH, thereby influencing the measurement. To minimize the effect of these factors, additional precautions were taken. Most important is the use of crude extract containing the expression vector without insert as negative control. To further reduce the likelihood of false positives, a time limit for nitrilase reaction was used. Based on the control reactions observed with the well-characterized *R. rhodochrous* K22 nitrilase (Kobayashi et al. 1990; Kobayashi et al. 1992b), these thresholds were set to 15 min and an absorbance decrease at 340 nm of at least 0.75.

Many of the 70 putative metagenome-derived nitrilase candidate genes were not considered for functional verification. Reason for exclusion was redundancy or problems during expression. In the end, 13 putative nitrilases were screened against 9 different nitriles with 6 of them showing activity for at least one of the nitriles. As nitrilases are known to cluster based on their substrate spectrum (Howden and Preston 2009), the observed substrate specificity was further controlled by calculation of a phylogenetic tree consisting of the 6 active enzymes and characterized nitrilases. The specificity of Nit09 was underlined by its clustering with arylacetonitrilases, as its substrate phenylacetonitrile converted in the initial screening belongs to this class of chemicals. In contrast, the fumaronitrile-specific nitrilase Nit60 contributed to a branch with β-cyano-l-alanine nitrilases. To our knowledge, the three β-cyano-l-alanine nitrilases have not been tested with fumaronitrile or other dinitriles (Howden et al. 2009; Acera et al. 2017), while our test substrates did not contain β-cyano-l-alanine. Thus, we can only assume that Nit60 would show activity with this compound and that the β-cyano-l-alanine nitrilases harbor a so far unknown ability to degrade dinitriles. Furthermore, a cluster consisting of four succinonitrile- and fumaronitrile-degrading nitrilases and a characterized nitrilase from *Synechocystis* sp. PCC6803 was identified. While the PCC6803-derived nitrilase barely acts on mononitriles, it is highly active for fumaronitrile (Heinemann et al. 2003), further supporting the results of the high-throughput assay. We could show that a combination of targeted sequence data analyses with a high-throughput activity assay assists in the fast identification of functional nitrilase candidates. Another possible application of the high-throughput activity assay is to employ it for the rapid substrate spectrum determination of known or recovered active nitrilases with a variety of different nitriles. The subsequent isolation and verification of the phenylacetonitrile-degrading nitrilase finally proved the reliability of the novel nitrilase screening workflow, which provides a way to successfully narrow down bioinformatically identified enzyme candidates to the interesting functional representatives.

### A novel and stable arylacetonitrilase

Arylacetonitrilases (EC 3.5.5.5) are a rare group of enzymes, and to our knowledge, only 12 bacterial nitrilases of this type have been characterized (Table 5). Thus, the phenylacetonitrile-degrading enzyme Nit09 was chosen for further validation of the workflow, as its characterization also contributes to the knowledge on arylacetonitrilases. According to sequence similarity, *nit09* is affiliated to *Variovorax boronicumulans* (Table 1). Nitrile-degrading abilities of this species have been described previously, but phenylacetonitrile degradation has not been mentioned (Nielsen et al. 2007; Zhang et al. 2012; Egelkamp et al. 2017; Sun et al. 2018). The molecular mass of Nit09 (36 kDa) is close to molecular masses of other known arylacetonitrilases such as the enzymes from *Pseudomonas* sp. UW4 (33 kDa; Duca et al. 2014), *Bradyrhizobium japonicum* USDA110 (37 kDa; Zhu et al. 2007a), or *P. fluorescens* EBC191 (38 kDa; Kiziak et al. 2005). In contrast, the broad pH range of Nit09 activity is not that common for nitrilases of this type, including the enzymes from *Alcaligenes faecalis* JM3 (Nagasawa et al. 1990) and *A. faecalis* ATCC 8750 (Yamamoto et al. 1992). In addition, Nit09 exhibits an unusual long-term stability with 80% remaining activity after 3 weeks of incubation at 4 °C; in contrast, the arylacetonitrilases from *A. faecalis* JM3 and *P. fluorescens* EBC191 lose up to 80% activity in 10 days.

**Table 5:** Properties of characterized bacterial arylacetonitrilases

Organism	pH optimum	Temperature optimum (°C)	Molecular mass (kDa)	Accession number	Reference
*Alcaligenes faecalis* JM3	7.5	45	44	BAA02684	Nagasawa et al. (1990)
*Alcaligenes faecalis* ATCC 8750	7.5	40–45	32	CUI34632	Yamamoto et al. (1992)
*Alcaligenes faecalis* ZJUTB10	7.5	40	44	AEP34036	Liu et al. (2011)
*Alcaligenes* sp*.* ECU0401	8.0	40–45	39	ACS13754	Zhang et al. (2011b)
*Alcaligenes* sp. MTCC 10675	6.5	50	60	AGC11817	Bhatia et al. (2014)
*Bradyrhizobium diazoefficiens* USDA110	–	–	37	NP_773042	Zhu et al. (2007a)
*Burkholderia cenocepacia* J2315	8.0	45	37	CAR52890	Wang et al. (2013)
*Burkholderia xenovorans LB400*	–	–	39	YP_559838	Seffernick et al. (2009)
*Luminiphilus syltensis* NOR5-1B	7.0	40	43	EED35210	Sun et al. (2015)
*Pseudomonas fluorescens* EBC191	6.5	50	38	AAW79573	Kiziak et al. (2005)
*Pseudomonas putida* MTCC 5110	7.0	40	43	ABV21758	Banerjee et al. (2006)
*Pseudomonas* sp. UW4	6.0	50	33	AFY19658	Duca et al. (2014)
*Variovorax boronicumulans* (predicted)	6.0	50	36	MN689843	This study

During initial screening, minor degradation of other nitriles was observed in the crude extract containing Nit09. None of these substrates was degraded by the purified enzyme, underlining the importance of the 15-min threshold to avoid identification of false positive candidates.

Additional tests with substrates structurally related to phenylacetonitrile provided a deeper insight into specificity of the nitrilase. Weak activity on non-arylacetonitriles was only recorded for 3-phenypropionitrile, demonstrating the importance of an acetonitrile-like residue attached to the aryl structure for catalytic function. Halogenic ortho, meta, and para substitutions at the benzyl residue led compared with phenylacetonitrile as substrate to a strong reduction of enzymatic activity. This behavior is not common for other arylacetonitrilases such as the enzymes from *A. faecalis* JM3 (Nagasawa et al. 1990), *A. faecalis* ATCC 8750 (Yamamoto et al. 1992), and *P. putida* MTCC 5110 (Banerjee et al. 2006), as in all tested cases at least one type of substitution led to increased activity. Interestingly, 1,4-phenylenediacetonitrile was converted with 19% relative activity, indicating an interference of halogens with the active center. Reduced activity was also observed for the plant hormone predecessor 3-indoleacetonitrile. The larger aromatic structure seems to cause general steric problems, as this is also the case for other arylacetonitrilases such as the enzymes from *A. faecalis* JM3 (Nagasawa et al. 1990), *Burkholderia cenocepacia* J2315 (Wang et al. 2013), *P. fluorescens* EBC191 (Kiziak et al. 2005), and *P. putida* MTCC 5110 (Banerjee et al. 2006) exhibited reduced activity with this compound. In contrast, the heteroaromatic structure found in thiophene results in increased enzymatic activity compared to phenylacetonitrile, probably due to the assistance of the sulfur in the nucleophilic attack performed by nitrilases (Ramteke et al. 2013). Furthermore, an effect of the position of the heteroatom was recorded. 2-Thiopheneacetonitrile was converted more than twice as efficient as 3-thiopheneacetonitrile by Nit09. This strong influence of the heteroatom is typical for arylacetonitrilases and can lead to more than a tenfold difference in enzymatic activity (Nagasawa et al. 1990; Yamamoto et al. 1992; Kiziak et al. 2005).

Nit09 was strongly inhibited by the thiol-specific reagents AgNO_3_ and HgCl_2_. Most likely, thiol groups build an important part of the active site, which is in line with the proposed Cys-Glu-Lys catalytic triad of nitrilases (Fernandes et al. 2006). Most arylacetonitrilases such as the enzymes from *A. faecalis* JM3 (Nagasawa et al. 1990), *A. faecalis* ATCC 8750 (Yamamoto et al. 1992), and *A. faecalis* ZJUTB10 (Liu et al. 2011) are also inhibited by other heavy metal ions like Cu^2+^ or Co^2+^, whereas the activity of Nit09 was almost unaffected by the presence of these ions. Interestingly, an increased activity was detected in the presence of ZnCl_2_. In general, nitrilase activities are independent of metal cofactors (Gong et al. 2012). This was supported by the lack of Nit09 activity inhibition by the presence of the chelating agents EDTA and sodium azide. Thus, a different mechanism like enhanced protein stability in the presence of Zn^2+^ might explain the detected activity increase of Nit09.

The characterization of arylacetonitrilase Nit09 provided additional knowledge on this rare group of nitrilases. The substrate range is common for this enzyme type, but the combination of a low pH optimum, a high temperature optimum, and an unusual long-term stability with a pronounced resistance against most metal ions is exceptional. Arylacetonitrilases are industrially interesting biocatalysts as they are currently used for the large-scale conversion of mandelonitrile to mandelic acid (Gong et al. 2012). Thus, identification of new enzymes of this type might open new production routes. Interesting in this respect is the conversion of phenylacetonitrile to phenylacetic acid, which is used among others for the production of penicillin G (Ziemons et al. 2017). The new insights obtained by identification and analysis of Nit09 contribute to the understanding of the sequence function relationship of arylacetonitrilases and thus to the development of better or novel nitrilase biocatalysts.

## Electronic supplementary material

This article contains supplementary material. The supplementary data files (Data File S1, Data File S2 and Data File S3), Table S1 and Figure S1 are given in the following files: Supplementary Data_File_S1.fasta, Supplementary Data_File_S2.xlsx, Supplementary Data_File_S3.txt and Supplementary_Figure_S1_and_Table_S1.pdf.

ESM 1 (FASTA 39 kb)

ESM 2 (XLSX 56 kb)

ESM 3 (TXT 5 kb)

ESM 4 (PDF 220 kb)
