# Supplementary material for: From sequence to function: a new workflow for nitrilase identification
Source: Appl Microbiol Biotechnol. 2020 Apr 14;104(11):4957–70. doi: 10.1007/s00253-020-10544-9 (PMC7228900; doi:10.1007/s00253-020-10544-9)
Supplement: Supplementary file 4 — (PDF 220 kb) [file 253_2020_10544_MOESM4_ESM.pdf]

Applied Microbiology and Biotechnology

From sequence to function: a new workflow for nitrilase identification

Richard Egelkamp,[1*] Ines Friedrich,[1*] Robert Hertel,[1] Rolf Daniel[1#]

[1]Genomic and Applied Microbiology & Göttingen Genomics Laboratory, Institute of Microbiology and Genetics, Georg-August-University of Göttingen, Grisebachstraße 8, 37077 Göttingen, Germany

[*]Richard Egelkamp and Ines Friedrich contributed equally to this work.

[#]Correspondence:

Rolf Daniel

Genomic and Applied Microbiology & Göttingen Genomics Laboratory, Institute of Microbiology and Genetics, University of Göttingen, Grisebachstr. 8, 37077 Göttingen, Germany. Phone: +49-551-3933827, Fax: +49-551-3912181, Email: rdaniel@gwdg.de

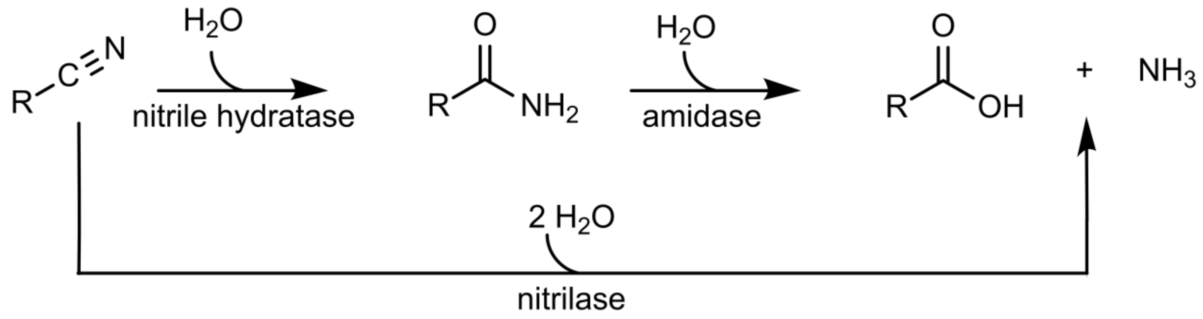

Fig. S1 Enzymatic nitrile degradation pathways.

Table S1 Primer list. T_m, melting temperature; Locustag, NCBI identifier of putative nitrilases; Restriction site, integrated restriction site for cloning into pBAD18; FW, forward primer; RV, reverse primer. Red/green/grey, restriction site; bold, stop codon; pink, hexahistidine sequence; blue, ribosomal binding site; orange, spacer sequence; small letters, bracketing sequence; normal letters, binding sequence.

Primer	Sequence	T_m	Locustag	Restriction sites	Direction
IF001	ataGAATTCAGGAGGTACTAGATGACTACTCAACAGCATCGTGTAC	56	D1872_207190	EcoRI	FW
IF002	tatTCTAGAgctc**CTA**TGCCAAAGGTGTTGGTTTCC	55	D1872_207190	XbaI	RV
IF004	tatTCTAGAgctc**CTA**ACCCATCACCTCTTCCAGC	57	D3C71_946180<br>D3C72_569880	XbaI	RV
IF005	ataGAATTCAGGAGGTACTAGATGGCCAAATCAATTGTTGCTGCG	56	D3C71_39170	EcoRI	FW
IF006	tatTCTAGAgctc**TTA**TGTGTTGAAACGCACCCCAG	55	D3C71_39170	XbaI	RV
IF007	ataGAATTCAGGAGGTACTAGATGACGATCTTCAAGGTAGCGG	55	D3C71_46180	EcoRI	FW
IF008	tatTCTAGAgctc**TTA**TTTGTTCTGCTCCATAGTGTC	52	D3C71_46180	XbaI	RV
IF009	ataGGTACCAGGAGGTACTAGATGAAGGAATATCCAAAATTCAAGGCAG	56	D3C71_81030	KpnI	FW
IF010	tatTCTAGAgctc**TTA**TTGCAAACGGTCGCTGCC	54	D3C71_81030	XbaI	RV
IF011	ataGAATTCAGGAGGTACTAGATGTCCGTGCATCAGCGTTTCAAGG	59	D3C71_205410	EcoRI	FW
IF012	tatTCTAGAgctc**TCA**GGCCTCCTGCGGGCCGG	62	D3C71_205410	XbaI	RV
IF014	tatTCTAGAgctc**TTA**CCGAACGACTTCTTCCGTC	55	D3C72_306600	XbaI	RV
IF015	ataGAATTCAGGAGGTACTAGATGACACGCCTCGCCGCCAG	60	D3C71_573540	EcoRI	FW
IF016	tatTCTAGAgctc**TTA**GATCGCAAATTGCTCTGGCCAG	58	D3C71_573540	XbaI	RV
IF017	ataGAATTCAGGAGGTACTAGATGCCGACCACCGTCCACCC	60	D3C71_601570	EcoRI	FW
IF018	tatTCTAGAgctc**TCA**GGCCACGACCGGCTCGG	62	D3C71_601570	XbaI	RV
IF019	ataGAATTCAGGAGGTACTAGATGCCCAAGTCAATCGTTGCTG	55	D3C71_775930<br>D3C72_447890	EcoRI	FW
IF020	tatTCTAGAgctc**TTA**TGTGTTGAAGCGCACACCG	55	D3C71_775930	XbaI	RV
IF021	ataGAATTCAGGAGGTACTAGATGACCAAGATAGCCATCATTCAG	54	D3C71_804950<br>D3C75_581680<br>D3C73_340990<br>D3C72_665010	EcoRI	FW
IF022	tatTCTAGAgctc**TCA**CTCGTCGATGAAGCGCA	54	D3C71_804950<br>D3C75_581680	XbaI	RV

IF023	ataGAATTCAGGAGGTACTAGATGACCCTCGTCAAAGCCGC	56	D3C71_946180<br>D3C72_569880	EcoRI	FW
IF024	ataGAATTCAGGAGGTACTAGATGCCCAAATCAATTGTTGCGGC	55	D3C71_1270450<br>D3C75_459950	EcoRI	FW
IF025	tatTCTAGAgctc**TCA**GGACGTGAAGCGCACCC	58	D3C71_1270450	XbaI	RV
IF029	tatTCTAGAgctc**CTA**AATGCCGGCTTCGCTCTT	54	D3C75_279060	XbaI	RV
IF033	tatTCTAGAgctc**TTA**GCTATTTTGAATAGCGTCGTAA	51	D3C75_211040	XbaI	RV
IF038	ataGAATTCAGGAGGTACTAGATGAAGGAATACCCAAAATTCAAGG	53	D3C72_113100	EcoRI	FW
IF039	tatTCTAGAgctc**TCA**CTGCAGGCGATCACTAC	54	D3C72_113100	XbaI	RV
IF040	ataGAATTCAGGAGGTACTAGATGCGCCAACACCACGTCGC	58	D3C72_226320	EcoRI	FW
IF041	tatTCTAGAgctc**TCA**GTCGCTGCGCGTGGTTAG	58	D3C72_226320	XbaI	RV
IF042	ataGAATTCAGGAGGTACTAGATGATGAAGGGGAGGGTCGTC	56	D3C72_260430	EcoRI	FW
IF043	tatTCTAGAgctc**TCA**AACAGCATGGTCGTCGTCC	57	D3C72_260430	XbaI	RV
IF044	ataGAATTCAGGAGGTACTAGATGCGTCAGCACCGTGTCGC	58	D3C72_369090	EcoRI	FW
IF045	tatTCTAGAgctc**TCA**ATCGCTCAAGGTGGTCAGAAC	57	D3C72_369090	XbaI	RV
IF046	tatTCTAGAgctc**TCA**TGTGTTGAAACGGACACCGG	57	D3C72_447890	EcoRI	RV
IF047	ataGGTACCAGGAGGTACTAGATGGAGTTCCCTAAATTCAAAGC	52	D3C72_512120	KpnI	FW
IF048	tatTCTAGAgctc**TTA**TTTTTTATGAAACTCCTCATGAGTA	51	D3C72_512120	XbaI	RV
IF049	tatTCTAGAgctc**TCA**CCCGTCGATATAGCGCA	54	D3C72_665010	XbaI	RV
IF050	ataGAATTCAGGAGGTACTAGATGTCACAGAAACGCATCGTGC	55	D3C72_810700	EcoRI	FW
IF051	tatTCTAGAgctc**TCA**GTCATCAACTGCCGGATC	54	D3C72_810700	XbaI	RV
IF052	ataGGTACCAGGAGGTACTAGATGATCCGCCTGGCGGCCTG	60	D3C72_823800	KpnI	FW
IF053	tatTCTAGAgctc**TCA**CAGGACTCTGTCAAACTGCTCC	59	D3C72_823800	XbaI	RV
IF054	ataGAATTCAGGAGGTACTAGATGCGCCACATCGCGCTGGAAGG	62	D3C72_995360	EcoRI	FW
IF055	tatTCTAGAgctc**TCA**AGGCTCCTGCGCGCCGC	62	D3C72_995360	XbaI	RV
IF056	ataGAATTCAGGAGGTACTAGATGAACGAGCGCGACCACGG	58	D3C72_1253310	EcoRI	FW
IF057	tatTCTAGAgctc**TCA**GTCATCAATTGCCGGCTCGAAG	59	D3C72_1253310	XbaI	RV
IF058	ataGAATTCAGGAGGTACTAGATGCACGTGGCCAGCTATATC	54	D3C72_1868530	EcoRI	FW
IF059	tatTCTAGAgctc**TTA**CTTGCTCTCGGAAAGGTCGA	55	D3C72_1868530	XbaI	RV
IF060	tatTCTAGAgctc**TCA**CTCGTCGATGAGGCGCA	56	D3C73_340990	XbaI	RV
IF061	ataGAATTCAGGAGGTACTAGATGACCATCGTCAAAGCCGC	54	D3C73_384910	EcoRI	FW
IF062	tatTCTAGAgctc**CTA**CCCAATCACTTCTTCCATCC	55	D3C73_384910	XbaI	RV

			D3C75_831420		
IF063	ataGAATTCAGGAGGTACTAGATGCCCAAATCAATCGTTGCGG	55	D3C73_406050	EcoRI	FW
IF064	tatTCTAGAgctc**TCA**GGAAGTGAAGCGCACCC	56	D3C73_406050<br>D3C75_459950	XbaI	RV
IF065	ataGAATTCAGGAGGTACTAGATGCCCAAGTCAATCGTTGCCG	57	D3C73_579180	EcoRI	FW
IF066	tatTCTAGAgctc**TCA**GGAGGTGAAGCGCACCC	58	D3C75_348960<br>D3C73_579180	XbaI	RV
IF067	ataGAATTCAGGAGGTACTAGATGTCTCAGTCCTACAAGGCG	54	D3C73_852800	EcoRI	FW
IF068	tatTCTAGAgctc**TCA**AACAGCAAGATCGTCCTC	52	D3C73_852800	XbaI	RV
IF069	ataGGTACCAGGAGGTACTAGATGAAACAAGCTATCAAGGTTGCCT	54	D3C73_958560	KpnI	FW
IF070	tatTCTAGAgctc**TTA**TAATTCTTGAAAGTGCACCGCT	53	D3C73_958560	XbaI	RV
IF071	ataGAATTCAGGAGGTACTAGATGAATCAAAACTTCAAAGCCGC	52	D3C75_211040	EcoRI	FW
IF072	ataGGTACCAGGAGGTACTAGATGTCCAATTATCCCAAATACCGAG	54	D3C75_279060	KpnI	FW
IF073	ataGAATTCAGGAGGTACTAGATGCCCAAATCAATTGTCGCGGC	57	D3C75_348960	EcoRI	FW
IF074	ataGAATTCAGGAGGTACTAGATGGCCGATGGCGAACAGAT	54	D3C75_831420	EcoRI	FW
IF075	ataGAATTCAGGAGGTACTAGATGGTGTATACGACATTTAAGCCT	52	D3C72_306600	EcoRI	FW
IF076	ataGAATTCAGGAGGTACTAGATGTC	55	K22 nitrilase	EcoRI	FW
IF077	tatTCTAGAgctc**TTA**AGCTTCAGCT	55	K22 nitrilase	XbaI	RV
IF078	ataGAATTCAGGAGGTACTAGATGCATCATCACCACCACCATTCTTCTAACCCGGAACTGAAATACAC	55	K22 nitrilase	EcoRI	FW
IF079	tatTCTAGAgctc**TTA**ATGGTGGTGGTGATGATGAGCTTCAGCTTTAGCACGACCA	55	K22 nitrilase	XbaI	RV
IF091	ataGAATTCAGGAGGTACTAGATGCATCATCACCACCACCATCCGACCACCGTCCACCC	60	D3C71_601570	EcoRI	FW
IF092	tatTCTAGAgctc**TCA**ATGGTGGTGGTGATGATGGGCCACGACCGGCTCGG	62	D3C71_601570	XbaI	RV
